# The potential impact of melanosomal pH and metabolism on melanoma

**DOI:** 10.3389/fonc.2022.887770

**Published:** 2022-11-22

**Authors:** Jaewon You, Maftuna Yusupova, Jonathan H. Zippin

**Affiliations:** Department of Dermatology, Weill Cornell Medical College of Cornell University, New York, NY, United States

**Keywords:** melanoma, melanosome, pH, reactive oxygen (ROS), melanin, cAMP

## Abstract

Melanin is synthesized in melanocytes and is transferred into keratinocytes to block the effects of ultraviolet (UV) radiation and is important for preventing skin cancers including melanoma. However, it is known that after melanomagenesis and melanoma invasion or metastases, melanin synthesis still occurs. Since melanoma cells are no longer involved in the sun tanning process, it is unclear why melanocytes would maintain melanin synthesis after melanomagenesis has occurred. Aside from blocking UV-induced DNA mutation, melanin may provide other metabolic functions that could benefit melanoma. In addition, studies have suggested that there may be a selective advantage to melanin synthesis in melanoma; however, mechanisms regulating melanin synthesis outside the epidermis or hair follicle is unknown. We will discuss how melanosomal pH controls melanin synthesis in melanocytes and how melanosomal pH control of melanin synthesis might function in melanoma. We will also discuss potential reasons why melanin synthesis might be beneficial for melanoma cellular metabolism and provide a rationale for why melanin synthesis is not limited to benign melanocytes.

## Canonical regulation of melanogenesis

**Figure 1:**
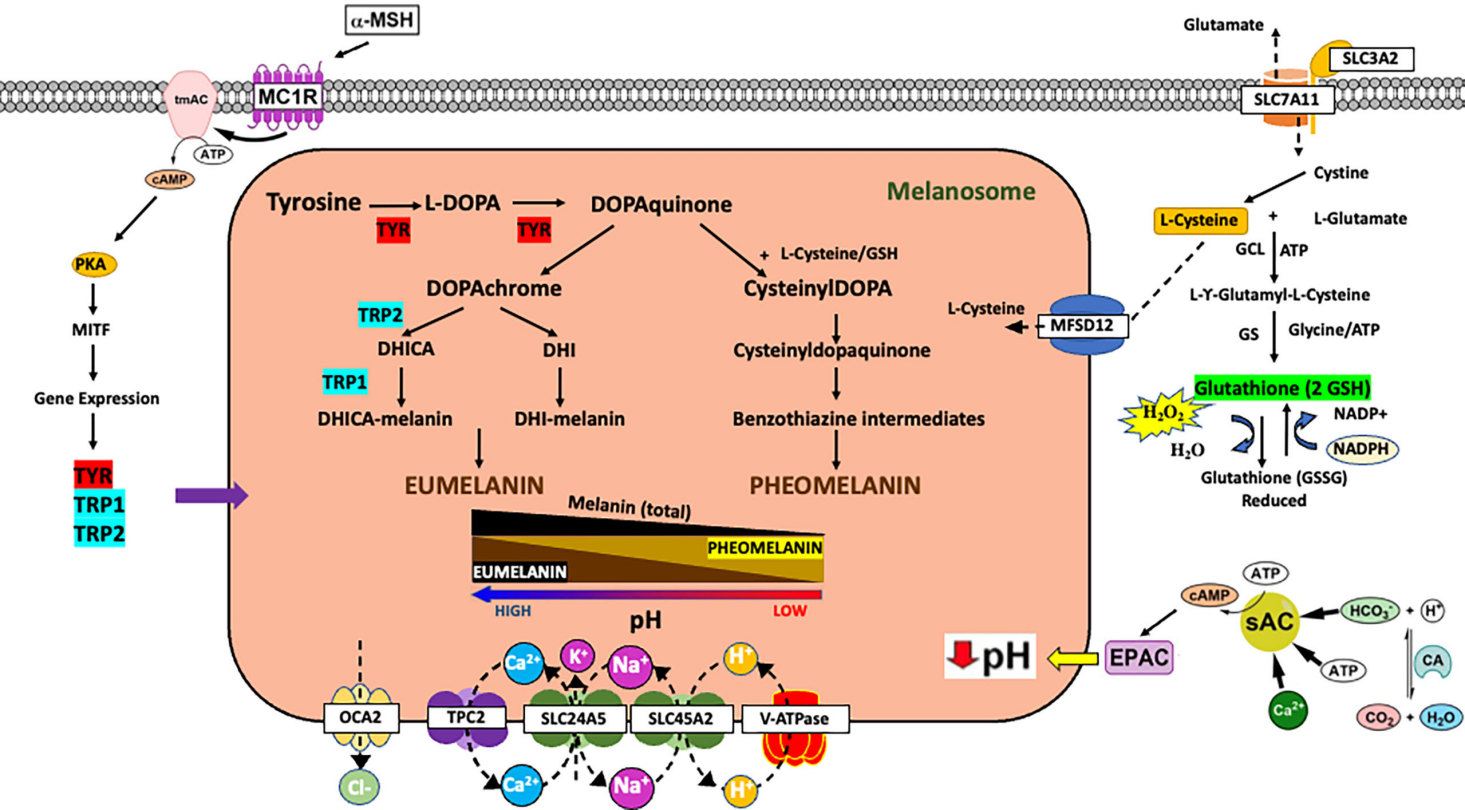
Impact of melanin metabolism on oxidative stress in melanoma. Overview of mechanisms of controlling melanin synthesis *via* gene expression or control of melanosome pH. Schematic of tyrosine metabolism in the cell highlighting the impact of melanin metabolism on oxidative stress. a-MSH, alpha melanocortin stimulating hormone; MC1R, melanocortin 1 receptor; tmAC, transmembrane adenylyl cyclase; PKA, protein kinase A; TYR, tyrosinase; TRP1, TYRP1/tyrosinase related protein 1; TRP2, TYRP2/DCT/tyrosinase related protein 2; EPAC, exchange protein activated by cAMP; sAC, soluble adenylyl cyclase; CA, carbonic anhydrase.

Ultraviolet (UV) radiation leads to numerous effects on distinct cells within the skin including keratinocytes, melanocytes, and fibroblasts. Many of the effects of UV lead to the release of paracrine factors which have both local and systemic effects (1). Melanin synthesis is an evolutionarily conserved metabolic process that has evolved in higher organisms to protect tissues from UV radiation (2). There are two types of melanin in the skin: eumelanin and pheomelanin (3). Both types of melanin are synthesized from the amino acid tyrosine *via* both enzymatic and non-enzymatic reactions (**Figure 1**) (4). Pheomelanin is favored when the melanin synthetic enzymes TYRP1/TRP1 and TYRP2/TRP2/DCT have low activity since pheomelanin is largely synthesized *via* non-enzymatic reactions and mainly relies on the concentration of dopaquinone and cysteine (**Figure 1**) (5–8). Upon the upregulation of TYRP1 and TYRP2, dopaquinone is diverted to eumelanin. High eumelanin is thought to be protective and high pheomelanin is associated with carcinogenesis (9). This is because eumelanin absorbs UV light and reactive oxygen species (ROS) whereas pheomelanin consumes cysteine and can generate ROS on its own (**Figure 1**) (10–12). Therefore, the regulation of the eumelanin to pheomelanin ratio is important for the control of UV-induced DNA damage and metabolically produced ROS.

There are a variety of external stimuli and genetic factors that influence melanin synthesis (13, 14). First and foremost, melanin synthesis in the skin is associated with polymorphisms in numerous genes (15), which can impact both baseline melanin synthesis and the ability of the skin to respond to external signals such as UV radiation. In the canonical response of skin to UV radiation, keratinocytes in the skin respond to UV radiation induced DNA damage by secreting α-melanocyte-stimulating hormone (α-MSH) which activates the melanocortin 1 receptor (MC1R), a G protein-coupled receptor on the surface of melanocytes, leading to activation of a cAMP signaling cascade and an increase in eumelanin production (2) (**Figure 1**). The increase in eumelanin leads to photoprotection against UV-induced DNA damage, thereby lowering the risk of sun-induced carcinogenesis. The MC1R gene is known to be highly polymorphic in Caucasian populations, and those deficient in MC1R activity are characterized by red hair, fair skin, poor tanning, and an increased risk of melanoma and skin cancer. There are also other paracrine factors and hormones that can impact melanin synthesis by altering intracellular cAMP (14). However, there are other genes critical for skin pigmentation which encode for proteins that do not control pigmentation *via* signal transduction cascades but instead have a defined function at the melanosome, the organelle responsible for making melanin (16). For example, polymorphisms in genes such as *OCA2*, *SLC45A2*, and *TPC2* affect baseline pigmentation and when the proteins they encode are non-functional, diseases of pigmentation such as albinism occur (17, 18). The proteins encoded by these genes are membrane channels and are present on the melanosome. These proteins mainly function to control melanosome pH (4). Melanosomal pH regulates melanin synthesis because tyrosinase, the rate limiting enzyme in melanin synthesis, is pH sensitive (**Figure 1**) (4, 19–21). Tyrosinase requires an acidic pH for the hydroxylation of tyrosine into L-DOPA (20). Once L-DOPA is formed it can act as a co-factor for additional tyrosine hydroxylation. Thus, tyrosinase can be active at low pH; however, L-DOPA oxidation is favored in more alkaline pH. Therefore, overall melanin synthesis is accelerated as melanosome pH approaches neutral (19, 22, 23). It should further be noted that under circumstances where complete tyrosine hydroxylation and/or L-DOPA oxidation is not possible these metabolites have the potential to be released and possibly induce inter- and intracellular biology (24–26). Polymorphisms and loss of function mutations in these genes are associated with skin cancer and melanoma risk most likely due to a reduction in eumelanin synthesis (27, 28). In addition to melanosome proteins important for the control of melanosome pH, there are other melanosome proteins critical for cysteine (e.g., MFSD12) and metal ion import/export that have the potential to impact both melanin synthesis and global cellular metabolism. It is also important to note that melanosomal pH may be reflective of intracellular pH which is impacted by changes in extracellular pH. Extracellular pH varies greatly in the epidermis with the cornified layer having a recorded pH of 5.0 at the surface of the skin and a range of pHs from 6.3 to 6.9 at the stratum granulosum depending on the method used for pH measurement (29, 30). Regardless of measured pHs at the stratum granulosum, the proton concentration surrounding melanocytes is much higher than intracellular levels. Thus, the pH microenvironment around the melanocyte may also impact melanosomal pH and melanin synthesis.

## Non-canonical regulation of melanin synthesis *via* alteration of melanosomal pH

There are two cAMP pathways that can regulate melanogenesis: a canonical and a non-canonical pathway. In the canonical pathway, the activation of the melanocortin 1 receptor (MC1R) leads to the stimulation of the transmembrane adenylyl cyclase (tmAC) and production of cAMP which induces a gene expression program that increases the expression of *MITF, TYR, TYRP1*, and *TYRP2*; genes critical for melanogenesis (31, 32). In contrast, there is a non-canonical cAMP signaling pathway in melanocytes that is defined by the soluble adenylyl cyclase (sAC). sAC activation acidifies melanosomal pH and inhibits tyrosinase activity; whereas inhibition of sAC leads to the elevation of melanosome pH and the activation of tyrosinase (**Figure 1**) (21, 23). Melanosomal pH directly affects melanin metabolism because the rate-limiting enzyme tyrosinase is pH-sensitive (**Figure 1**). Melanosomes, depending on the stage of development and other factors, have variable pHs (19). When melanosomes mature their intra-organellar pH increases from 5 to 6.8 and pigment production increases (33). In addition, melanosomes from white/fair skin tend to be relatively more acidic as compared to melanosomes from black/dark skin, which tend to have a more neutral pH (19, 34). In addition to affecting the activity of tyrosinase, melanosomal pH, in cooperation with cysteine levels, can control the EM to PM ratio (**Figure 1**) (5, 21, 22, 35–37). Alkaline melanosomal pH favors EM over PM formation and thus promotes an anti-oxidant environment. Changes in the extracellular concentration of cystine are known to increase eumelanin levels presumably due to a decrease in intracellular cysteine levels (38). However, the mechanisms that control cystine/cysteine levels to affect melanin synthesis are poorly understood. Mouse models suggest that the subtle gray phenotype occurs because of a mutation in *Slc7a11*, which encodes for the plasma membrane cystine/glutamate exchanger xCT (**Figure 1**) (39). In the absence of SLC7A11 activity, cystine is not transported into the cell leading to a decrease in PM synthesis with little to no effect on EM. Recently, it was determined that the MFSD12 (major facilitator superfamily domain-containing protein 12) is important for the import of cysteine/cystine into the melanosome (**Figure 1**) (40), which may explain why polymorphisms in MFSD12 are associated with darker pigmentation in mice and humans (41, 42). Thus, differences in melanosomal pH can have significant effects on melanin metabolism.

## Association between genetic defects affecting melanosomal pH and melanomagenesis

The association between MC1R polymorphisms and melanoma risk is well established and is explained by defects in UV-induced tanning and protective eumelanin pigmentation (2). However, there are several polymorphisms that affect melanosomal channels and transporters which alter melanosomal pH and increase the risk of melanoma (18). Oculocutaneous albinism type 2 (OCA2) is caused by mutations in the *OCA2* gene. Individuals with *OCA2* mutation are at a higher risk of developing UV-induced skin cancers including melanoma. The OCA2 channel is normally incorporated into melanosomes early during melanosome maturation, and functions to neutralize melanosomal pH for optimal tyrosinase function and eumelanin production (**Figure 1**) (43, 44). Therefore, genetic mutations of the OCA2 channel lead to acidic melanosomal pH, hypopigmentation due to decreased eumelanin synthesis, and increased risk for melanoma. The *SLC45A2* (solute carrier family 45 member 2) gene encodes a H^+^/sugar co-transporter protein on the melanosomal membrane (**Figure 1**). *SLC45A2* is required for melanosomes to progress from stage III to IV. Mutations in the *SLC45A2* gene have been associated with oculocutaneous albinism type 4, a condition characterized by strong melanin deficiency. It has also been found that the *SLC45A2* transporter expressed ectopically in HeLa cells localizes to lysosomes and plays a role in raising lysosomal pH. This suggests that *SLC45A2* expression in melanocytes can potentially plays a role in modulating melanosomal pH to support melanin production. According to genome-wide association studies on European populations, DNA variants at the *OCA2* and *SLC45A2* genes have been associated with increased susceptibility to cutaneous malignant melanoma (45). Two pore channel 2 (TPC2/TPCN2) is an ion channel not exclusively expressed on melanosomes or in melanocytes and is a voltage-independent cation channel that responds to ligands instead of membrane potential (17, 46–48). Unlike OCA2 and SLC45A2, loss of TPC2 activity leads to an increase in melanin synthesis (17, 43, 49, 50). TPC2 is not associated with pigment diseases but polymorphisms are associated with hair color and UV sensitivity mostly in Dutch and Icelandic individuals (51–53). Whereas proteins that control melanosomal pH can impact the risk of developing melanoma, these same proteins are also differentially expressed during invasion and metastasis (54, 55). Thus, it is possible that changes in melanosomal pH may impact melanin synthesis in metastatic melanoma.

## Potential roles of melanin during melanomagenesis

Whereas melanin in the skin has a clear role in skin cancer protection, it is unclear why melanoma cells would maintain the production of this molecule. Since melanin synthesis generates toxic intermediates and can be an energetic process, it stands to reason that melanoma maintains this metabolic process because melanin supports the growth of melanoma cells after transformation. Eumelanin (EM) possesses antioxidant properties and pheomelanin (PM) is pro-oxidant. Both pigment types are derived from the common precursor dopaquinone, which is formed from the oxidation of L-tyrosine by the enzyme tyrosinase (4) (**Figure 1**). EM scavenges free radicals produced from UV induced damage due to its paramagnetic, redox, and ion exchange antioxidant properties. These properties neutralize reactive oxygen species (ROS) (56–58). PM is a yellow to reddish-brown pigment produced when cellular L-cysteine binds with dopaquinone to produce cysteinyldopa isomers (**Figure 1**) (4). The oxidation of this thiol-dopa produces PM. During the production of PM ROS is generated which can lead to cellular damage (59).

Cellular ROS is normally controlled by the enzyme glutathione-S-transferase (GST), which catalyzes the binding of GSH with dopaquinone and glutathione reductase (GR), which reduces the oxidized GSH in cells to a usable form (**Figure 1**) (60). GSH is a multifunctional molecule and is the main antioxidant used by cells to neutralize ROS. GSH is a ubiquitous compound that consists of the amino acids cysteine, glycine, and glutamate. It has a biologically active sulfhydryl (SH) group, which allows it to neutralize ROSs such as H_2_O_2_ (61). GSH is a non-enzymatic antioxidant and an endogenous redox buffer that donates electrons to peroxidases (62). GSH is also involved in other biochemical pathways such as maintaining the SH groups of proteins and other molecules, serving as a coenzyme for certain enzymes, and during detoxification processes within cells (61). GSH can be regenerated by NADPH which reduces its oxidized form (62). Thus, any cellular stress that leads to movement of cysteine into GSH could decrease cellular concentrations of L-cysteine and induce a eumelanotic shift in melanogenesis (38, 63). Since EM protects against ROS and PM produces ROS (e.g., H_2_O_2_) the balance between these two melanin synthetic pathways would affect ROS levels during melanomagenesis (**Figure 1**) (64).

ROS can affect tumor biology *via* many distinct mechanisms. Examples include modifying DNA or upregulating nitric oxide synthase (NOS) synthesis (65). ROS produced as byproducts of enzymatic and non-enzymatic metabolic processes include superoxide radicals (O2^.-^), hydroxyl radicals (OH^.^), and hydrogen peroxide (H_2_O_2_). At high levels, these exemplary species lead to the oxidation of cellular lipids, proteins, and DNA inducing cellular damage and mutation important for melanoma initiation and progression (62, 66). In contrast, lower levels of ROS promote activation of vital signaling pathways such as cellular proliferation and survival (67).

Melanoma cells promoting EM production at the expense of PM production preserve GSH for cellular ROS reduction because PM production requires L-cysteine. However, the mechanisms by which melanoma might alter melanin synthesis are not well established. Of note, the production of PM in the presence of L-cysteine is increased around pH 5.8-6.3, while the generation of EM is suppressed at pH 5.8 (68). This suggests that there may be a role for melanosomal pH in the control of EM/PM ratio and GSH synthesis. By increasing melanosomal pH, melanocytes could increase antioxidant EM and preserve antioxidant GSH levels to combat the effects of ROS. Aside from regulating ROS, melanogenesis can impact melanoma by altering the expression of stress related genes such as HIF-1a and metabolic regulatory proteins such as GLUT-1 (69).

## Potential roles of melanin during melanoma metastasis

The presence of melanin in cutaneous melanomas is associated with a higher risk of metastasis, aggressiveness of cancer, and death (70–73). In individuals with uveal melanomas, differential melanin synthesis is associated with a higher risk of metastasis and death (74). Independent of its effects on growth, melanomas with high levels of melanin appear to attenuate the efficacy of radiotherapy; patients with amelanotic melanomas had longer survival times when compared to pigmented melanomas (70). Thus, these reports suggest that melanin synthesis may play an important role in melanoma well beyond tumor initiation.

Metastasizing melanoma can appear as gray-black or blue-black suggesting a robust eumelanin synthetic pathway. In addition, increased ROS in metastatic melanoma cells can prevent metastasis. Oxidative stress can impair cancer progression by suppressing protein translation (75). Epithelial melanoma cells upon invasion and metastasis require antioxidant protection to successfully travel in the bloodstream to form secondary tumors; therefore, balancing ROS is critical for metastasizing tumors (76).

To manage ROS, metastatic cells have developed alternative mechanisms to overcome the toxic effects of ROS, such as producing NADPH to regenerate GSH reserves. Melanotic melanoma cells contain high amounts of reduced GSH and glutathione-S-transferase (GST) in the cytosol as suggested by the increased activity of glutathione reductase in these cells as compared to amelanotic melanoma cells (77). It is reported that detached cancer cells will increase their glucose uptake and upregulate the pentose phosphate pathway as well as other metabolic pathways to generate more NADPH (78–80) for the regeneration of reduced GSH. Alternatively, melanoma can also increase reduced GSH reserves by increasing serine and glycine synthesis (81).

Melanogenesis would be an excellent mechanism for balancing the cell’s overall redox levels. To restore the cell’s redox balance, the melanotic melanoma cells could produce more GSH (77) which could be achieved by diverting melanin synthesis away from pheomelanin. In addition, it has been suggested that melanin possesses some immunosuppressive properties and the ability to have other paracrine signaling effects *via* the production of certain melanin intermediates such as DOPA (82–84). Furthermore, since melanin metabolism affects tyrosine levels, there is a potential impact of altered melanin metabolism on the production of tyrosine-derived signal molecules. In addition, there is a potential impact of melanosomal metabolism on the cellular microenvironment which might enhance melanoma mestastasis (85). Thus, balancing melanosomal metabolism may be a critical mechanism during melanoma progression.

## Conclusions

Melanin has an established role in the protection of the epidermis from UV radiation. However, melanin synthesis continues in melanoma following invasion and metastasis. Given the potentially toxic effects of melanin synthesis, it is unclear why melanoma would maintain this metabolic process. Melanoma is very sensitive to ROS during invasion and metastasis. Since melanin metabolism can affect ROS both positively and negatively, it is possible that melanoma cells harness melanin metabolism to balance ROS. In addition, it appears that melanin levels can affect the melanoma therapeutic response. Whereas traditional methods of regulating melanin metabolism (e.g., MC1R) are not significantly altered in melanoma, genes important for the regulation of melanosomal pH are altered in melanoma. We propose that altering melanosomal pH may be an effective mechanism for the regulation of melanin synthesis. Specifically, modulating melanosomal pH could alter the eumelanin to pheomelanin ratio which has a dramatic effect on cellular ROS. Currently there is a paucity of studies focused on understanding melanosome metabolism (23, 40). We predict that additional investigation of melanosomal pH and melanosome metabolism in melanoma may reveal new mechanisms that affect melanoma metastasis or therapeutic response.

## Author contributions

JY, MY, and JZ all contributed to the writing and editing of the manuscript. All authors read and approved the final manuscript.

## Funding

This study was funded by the National Institute of Arthritis and Musculoskeletal and Skin Diseases (NIAMS), grant number 1 R01 AR077664-01A1.

## Conflict of interest

The authors declare that the research was conducted in the absence of any commercial or financial relationships that could be construed as a potential conflict of interest.

## Publisher’s note

All claims expressed in this article are solely those of the authors and do not necessarily represent those of their affiliated organizations, or those of the publisher, the editors and the reviewers. Any product that may be evaluated in this article, or claim that may be made by its manufacturer, is not guaranteed or endorsed by the publisher.
